# Impedimetric Detection of Ammonia and Low Molecular Weight Amines in the Gas Phase with Covalent Organic Frameworks

**DOI:** 10.3390/s20051385

**Published:** 2020-03-03

**Authors:** Andrés Rodríguez, Elio Rico, Cesar Sierra, Oscar Rodríguez

**Affiliations:** 1 Grupo de Investigación en Macromolécula, Departamento de Química, Facultad de Ciencias, Campus Universitario, Universidad Nacional de Colombia-Sede Bogotá, Edificio 451, 111321 Bogotá, Colombia; anrodriguezca@unal.edu.co (A.R.); earicov@unal.edu.co (E.R.); casierraa@unal.edu.co (C.S.); 2 Grupo de Electroquímica y Termodinámica Computacional, Departamento de Química, Facultad de Ciencias, Campus Universitario, Universidad Nacional de Colombia-Sede Bogotá, Edificio 451, 111321 Bogotá, Colombia

**Keywords:** impedance, thin films, vapors amines, imine

## Abstract

Two Covalent Organic Frameworks (COF), named TFP-BZ and TFP-DMBZ, were synthesized using the imine condensation between 1,3,5-triformylphloroglucinol (TFP) with benzidine (BZ) or 3,3-dimethylbenzidine (DMBZ). These materials were deposited, such as films over interdigitated electrodes (IDE), by chemical bath deposition, giving rise to TFP-BZ-IDE and TFP-DMBZ-IDE systems. The synthesized COFs powders were characterized by Powder X-Ray Diffraction (PXRD), Fourier Transform Infrared spectroscopy (FT-IR), solid-state Nuclear Magnetic Resonance (ssNMR), nitrogen adsorption isotherms, Scanning Electron Microscopy (SEM), and Raman spectroscopy, while the films were characterized by SEM and Raman. Ammonia and low molecular weight amine sensing were developed with the COF film systems using the impedance electrochemical spectroscopy (EIS). Results showed that the systems TFP-BZ-IDE and TFP-DMBZ-IDE detect low molecular weight amines selectively by impedimetric analysis. Remarkably, with no significant interference by other atmospheric gas compounds such as nitrogen, carbon dioxide, and methane. Additionally, both COF films presented a range of sensitivity at low amine concentrations below two ppm at room temperature.

## 1. Introduction

Our ecosystem and human activities can release a great variety of gas compounds to the atmosphere, including greenhouse gases (carbon dioxide, methane, ozone, among others). Moreover, harmful gases such as nitrogen oxides and ammonia, and even some low molecular weight amines, above specific low concentrations can be dangerous for human health [1,2,3]. Notably, the ammonia concentration in the atmosphere has increased because this compound has been used indiscriminately as an essential raw material in multiple chemical industries; as a result, the global emission level of NH_3_ has been doubled in the last 40 years (50 million tons in recent years) [4]. High levels of ammonia present a severe threat to the sustainability of our ecosystem and human health [5]. Therefore, to detect ammonia is a vital function for controlling the over release/production of NH_3_ as much in environmental sectors as in medical diagnostics [6].

It is well known that detecting sensitive and selectively ammonia is a challenge. Up to now, several techniques have been used in commercial ammonia detectors, among them, metal–oxide gas sensors [7,8,9,10,11], conducting polymer analyzers, and detection optical methods [12,13,14,15,16,17]. However, these strategies exhibit some disadvantages associated with high temperatures and irreversible reactions causing poisoning and lack the selectivity or sensitivity. Nonetheless, in order to improve the gas sensing properties, some researchers have developed new approaches to increase the specific surface area and also using composite films [18]. In this way, chemosensors synthetized on interdigitated electrodes (IDE) have been used frequently. The IDEs allow increasing contact area between the sensing material and the electrodes [19]. In addition, IDEs facilitate the measurement of multiple electrochemical properties, such as capacitance, conductivity/resistance, impedance, etc., without sophisticated electronics [19,20]. The impedimetric gas sensors have lately received considerable interest owing to the increasing demand for monitoring harmful gases, and because of its good selectivity, fast detection, high sensitivity, and facile sample pretreatment [21]. Therefore, the impedimetric detection of ammonia has been previously reported [22,23], using polypyrrole coated zinc oxide and ion-exchanged Y zeolites.

Recent studies have shown that interdigitated electrodes can improve the conductive properties of materials, generally referred to as bad conductors, such as the Covalent Organic Frameworks (COFs) [24]. COFs are highly porous materials formed by organic molecules joined by covalent bonds (linkage) with another organic molecule (linkers) forming an infinite and repetitive arrangement into two-dimensional (2D) and three-dimensional (3D) networks [25,26,27]. These materials have a high surface area, and due to the strength of the covalent bond, they exhibit excellent stability in aqueous solution, acidic media, and also at high temperatures and corrosive environments [28]. These properties make COFs suitable for gas storage, energy storage, water treatments, cancer treatment, fuel cells, catalysts, and explosives sensors [29,30,31,32,33,34,35,36]. The synthesis of COFs can be carried out by several methods, including solvothermal, dropwise, microwave, sonochemistry, and mechanochemistry [37,38,39,40]. All these methods added to the large number of organic reactions which can be used in the synthesis of COFs, making the structural and topological diversity of these materials abundant [37]. Regarding the electrical properties of COFs, reports in the literature show that COFs have poor electrical conductivity, which can become a problem in the development of an electrochemical sensor. For this reason, some strategies have been developed to increase conductivity and improve electrical communication throughout the entire material [30], where a conductive polymer, such as PEDOT (poly 3,4-ethylenedioxythiophene), is synthesized inside the pores of the COF.

In this work, two COFs were synthesized through the condensation of primary amines with aldehydes by the dropwise/solvothermal method. These COFs were deposited as films over interdigitated gold electrodes. Afterward, using these COF-electrode systems, it was possible to detect ammonia and low molecular weight amines using an impedimetric technique. Noteworthy, there have not been reports using COF and electric impedance for the detection of ammonia. 

## 2. Materials and Methods

### 2.1. Materials

1,3,5-Triformylphloroglucinol (TFP) was synthesized according to previous reports under a Duff reaction of phloroglucinol with hexamethylenetetramine (HMTA) [41]. All other reagents were purchased from commercial sources and used without previous purification. Commercial interdigitated gold electrodes (IDEs) were purchased from DropSens (IDEAU200) and were used as transducers in the sensing experiments (ceramic substrate: L 22.8 × W 7.6 × H 0.7 mm, 8 × 2 digits, 200 µm electrode bands/gaps).

### 2.2. Synthesis of COFs

**Scheme 1:**
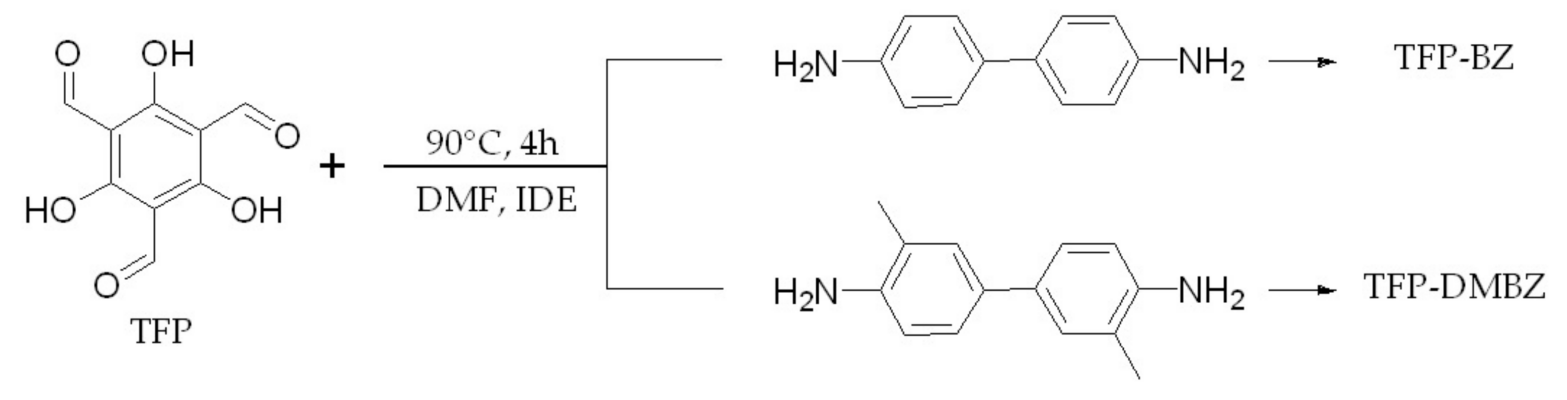
General conditions for the synthesis of Covalent Organic Frameworks (COFs) TFP-BZ and TFP-DMBZ over interdigitated electrodes (IDE).

COFs synthesis followed the thin layer protocol previously reported [42]. A 0.071 mmol amount of corresponding amine (benzidine (BZ) or 3,3-dimethylbenzidine (DMBZ)) were dissolved in 3.2 mL of *N*,*N*-dimethylformamide (DMF) in a glass vial. Into this vial was added a gold interdigitated electrode and magnetic bar, and then, the vial was capped and heated at 90 °C with low stirring. After the temperature was reached, 1 mL of TFP solution 10 mg/mL (0.048 mmol) was added slowly for 1 h. Subsequently, the reaction was carried out for 3 h (Scheme 1). Finally, the vial was open, and the modified electrodes with COF film on its surface and powder residues were separated. The COF films deposited over the electrodes, labeled as TFP-BZ-IDE and TFP-DMBZ-IDE, were washed with DMF, acetone, and distilled water three times. 

### 2.3. Characterization

The COFs powders residues from the synthesis over the electrodes were characterized by x-ray diffraction in an X’Pert Panalytical diffractometer with a copper anode (λ = 0.1506 nm), with a data acquisition time of 1 s, a range of 2–35 degrees 2θ, and a size step of 0.1 degrees. FT-IR was carried out on a Shimadzu IR prestige-21 with an attenuated total reflection (ATR) accessory in a range from 750 cm^−1^ to 4000 cm^−1^, 50 scans, and 4 cm^-1^ of resolution. The ssNMR experiments were recorded in a 400 MHz Bruker Avance III spectrometer with a 4 mm channel for solids (15 MHz). The data acquisitions for ^13^C used a combination between cross-polarization and magic angle spinning (CP/MAS) in a 7 mm rotor at room temperature. The N_2_ adsorptions were performed on an ASAP 2020 micrometrics instrument with 77 K nitrogen and 30 mg of sample approximately. On the other hand, for the COF films synthesized over the electrodes were characterized by SEM and Raman analysis, where the first was recorded on a JOEL JSM-6490L microscope with secondary electrons Everhaty–Thornley detector. The powders were cover with a Pt-Au 10 nm film approximately; the electron energy was 10 kV. The Raman experiments were carried out on a Thermo Scientific DXR with a laser excitation of 540 nm, 20 mW power, and 50× amplification.

### 2.4. Electrochemical Impedance Spectroscopy Experiments

The electrochemical impedance spectroscopy experiments were recorded on a Gamry Interface 1000 E potentiostat in potentiostatic mode at open circuit potential in the frequency range 1 Hz to 100 kHz with 10 mV amplitude of the test signal. The data were analyzed with the Gamry instrument version 7.05 package software.

### 2.5. Low Molecular Weight Amines Detection

The applicability of COFs as gas sensors for ammonia, methylamine, ethylamine, dimethylamine, and triethylamine detection was evaluated by the TFP-BZ-IDE and TFP-DMBZ-IDE systems. Low molecular weight amine detection was carried out on a modified system previously reported [43], using a closed chamber with a fan and an electrode holder equipped. TFP-BZ-IDE and TFP-DMBZ-IDE systems were introduced inside the chamber, and amine vapors were injected by a septum in an injection port, modifying the system atmosphere. 

To evaluate the gas response, the COF system’s impedance was measured in air and the presence of amines. The impedance response data reported for each concentration corresponds to the value at which the measurement was stabilized after three air/amine vapor injections cycles.

## 3. Results and Discussion

### 3.1. Powder Characterization

The COFs synthesis on IDEs followed the chemical bath deposition technique; methodology that also produced a supernatant powder. Here named as residuals COF powders. The thin films’ characterization was a difficult procedure, and after several attempts of that, it was decided to make an extensive characterization of this residual powder and take it as a comparison pattern for the characterization of the COF-electrode systems.

#### 3.1.1. Powder X-ray Diffraction 

**Figure 1:**
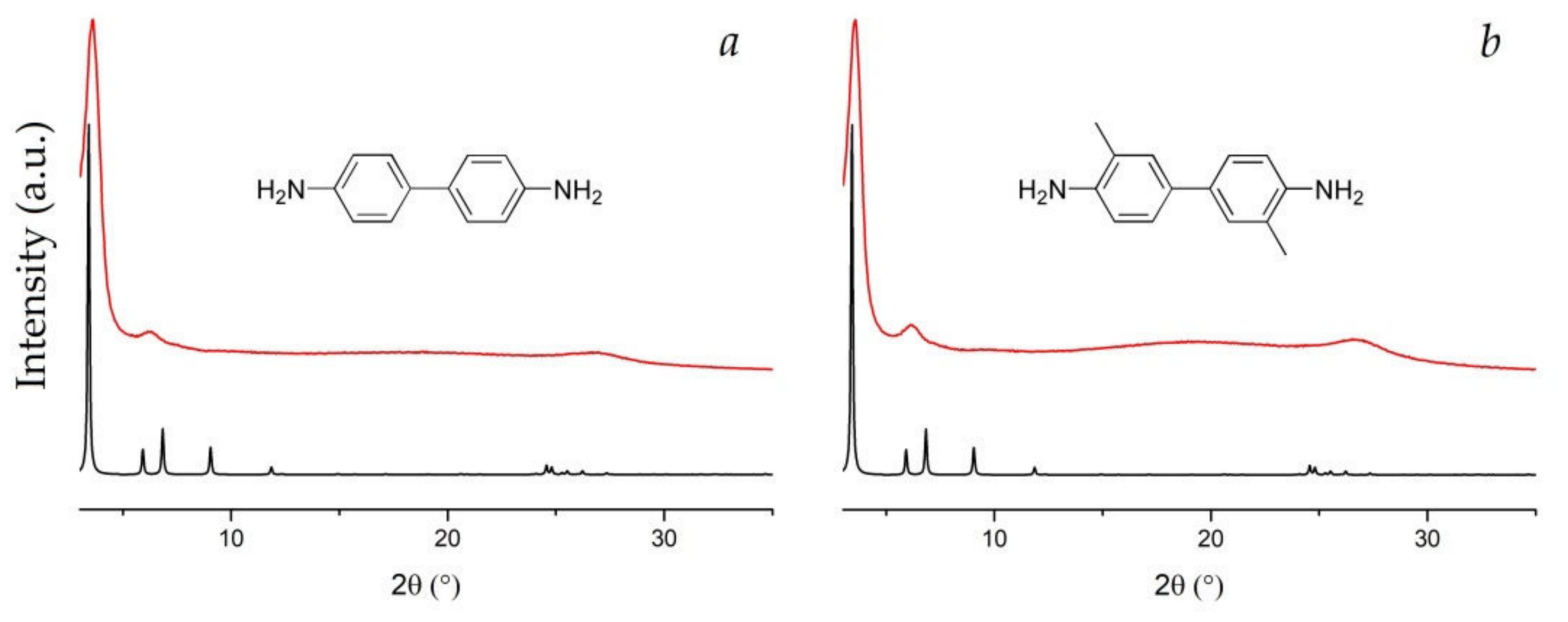
Experimental diffraction patterns (red) and simulated (blank) of (**a**) TFP-BZ and (**b**) TFP-DMBZ.

COFs showed a typical crystallinity for this kind of material exhibited a significant pick under 5° 2θ, exactly at 3.6° 2θ for both COFs (TFP-BZ and TFP-DMBZ) corresponding to the (100) plane and more picks at 6.2° 2θ and 26.6° 2θ corresponding to the (110) and (001) planes, respectively. The diffraction patterns are practically the same for both materials (Figure 1), since the chemical structures of the two COFs are very similar, as was reported previously [44].

#### 3.1.2. Fourier Transform Infrared Spectroscopy 

**Figure 2:**
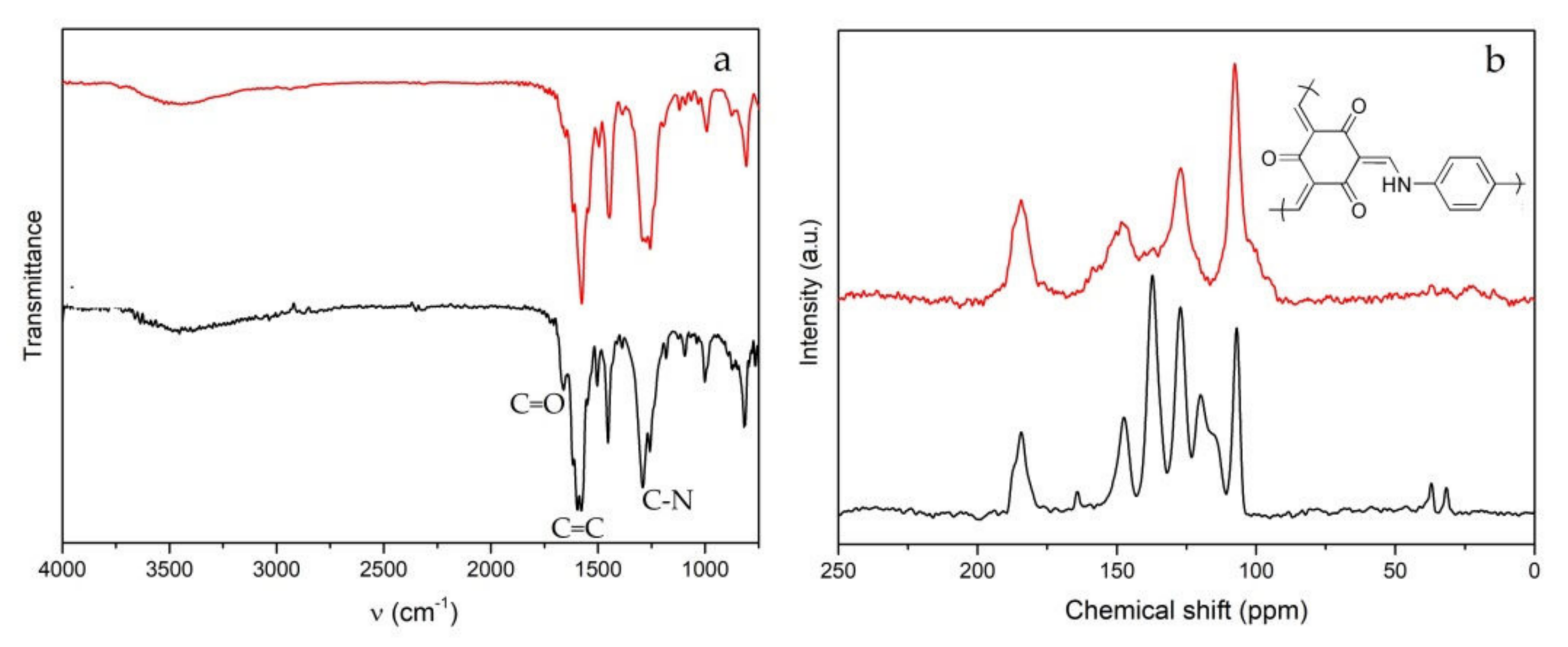
(**a**) FT-IR spectra and (**b**) ^13^C-ssNMR spectra for TFP-BZ (black) and TFP-DMBZ (red).

The FT-IR spectra of COFs present the typical broad and intensive bands correspondent to a characteristic C=C stretch for aromatics in the region of 1570 cm^−1^ (1582 cm^−1^ for TFP-BZ and 1576 cm^−1^ for TFP-DMBZ) and C-N stretch for amines near to 1260 cm^−1^ (1289 cm^−1^ and 1275 cm^−1^ for TFP-BZ and TFP-DMBZ, respectively). There are also weak bands close to 1650 cm^−1^ correspondent to C=O bonds of the keto form in the tautomerization keto-enamine form [28]. The disappearance of the N-H bonds (approximately at 3400 cm^−1^) and C=O bonds (1730 cm^−1^) shows that there are no precursor remnants (Figure 2a).

#### 3.1.3. Solid-State Nuclear Magnetic Resonance Spectroscopy 

The ssNMR results demonstrated that the keto form is predominant in the tautomerization keto-enamine form. The signals at 184.4 ppm for TFP-BZ and 184.1 ppm for TFP-DMBZ correspond to the carbonyl of the keto group (Figure 2b). Other characteristic signals of the keto form are those corresponding to sp^2^ carbon bonding with the nitrogen of amines at 107.0 ppm and 107.9 ppm for TFP-BZ and TFP-DMBZ, respectively (Figure 2b). 

#### 3.1.4. N_2_ Adsorption Isotherms

**Figure 3:**
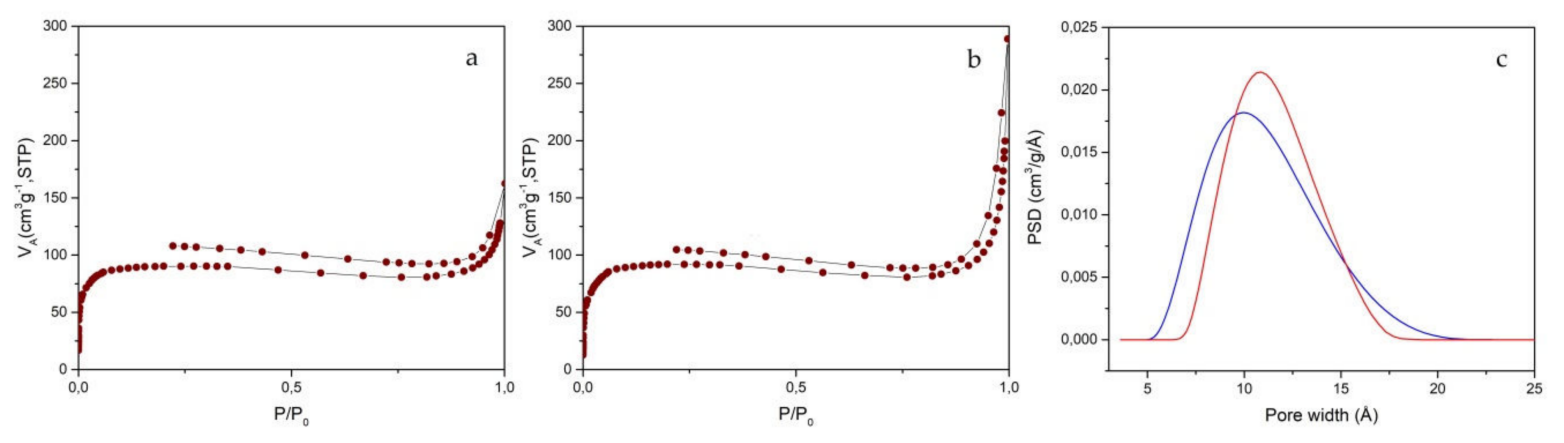
(**a**) N_2_ adsorption isotherm of TFP-BZ. (**b**) N_2_ adsorption isotherm of TFP-DMBZ. (**c**) Size pore distributions of TFP-BZ (blue) and TFP-DMBZ (red).

The nitrogen adsorption isotherms were studied to determine the COFs surface areas. The Brunauer–Emmett–Teller (BET) method was used to calculate the surface areas with a nonlocal density functional theory (NLDFT). The BET surface area was found to be 256.95 ± 8.39 m^2^ g^−1^ and 256.78 ± 9.83 m^2^g^−1^ for TFP-BZ (Figure 3a) and TFP-DMBZ (Figure 3b), respectively. The size pore distribution of both COFs showed maximums at 9.92 Å for TFP-BZ and 10.84 Å for TFP-DMBZ (Figure 3c), with isotherms showing important adsorptions in the regions of microporous (low relative pressures). The size pore distribution due to the population of width pore was below 20 Å, confirming the material microporosity.

#### 3.1.5. Scanning Electron Microscopy

**Figure 4:**
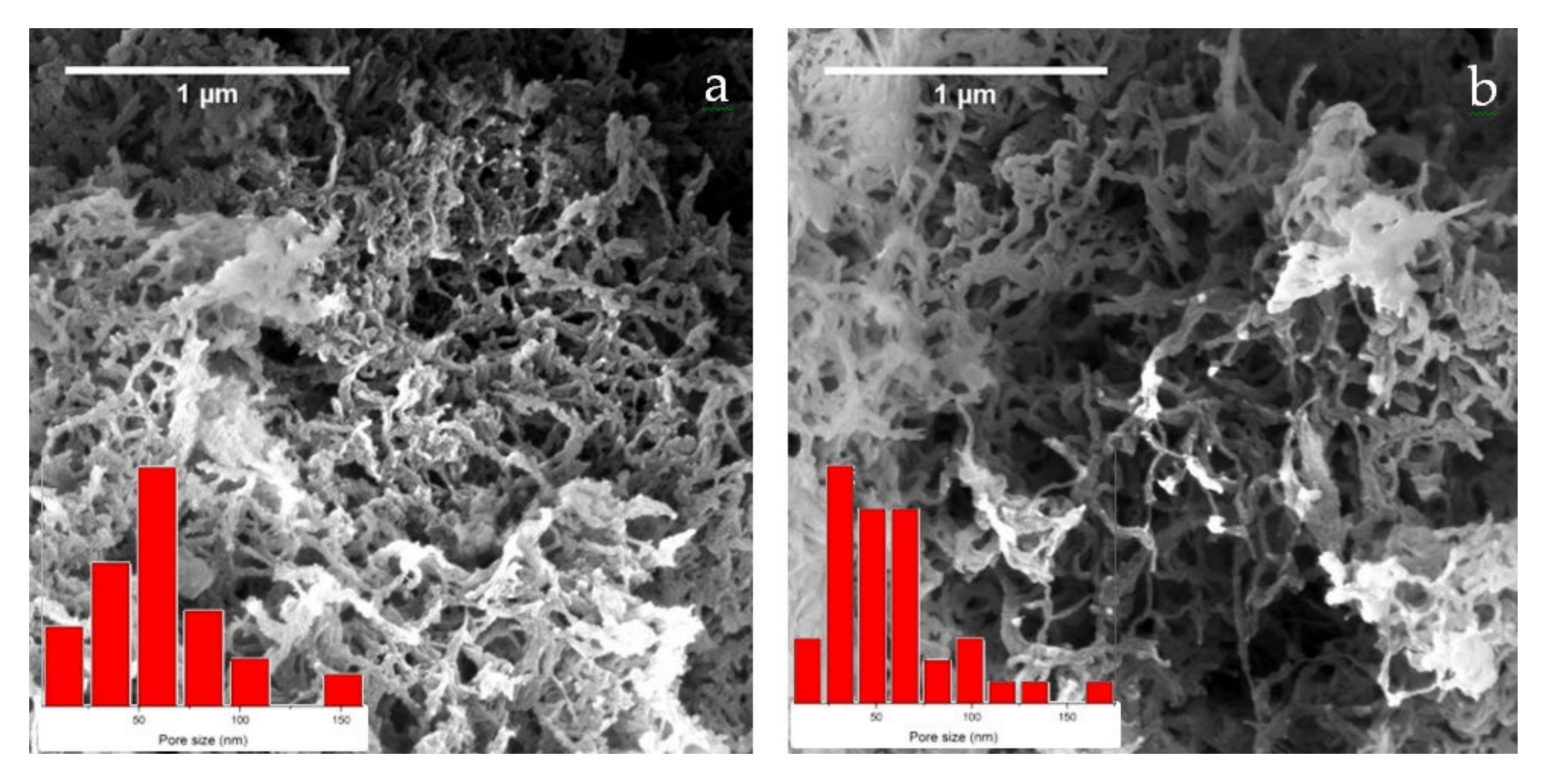
SEM images and pore size distribution of (**a**) TFP-BZ and (**b**) TFP-DMBZ.

The COFs morphologies by SEM images look like intercross wires forming a network with different kinds of pores. The morphology of TFP-BZ (Figure 4a) and TFP-DMBZ (Figure 4b) are similar, both with the presence of mesoporous and macroporous. The SEM micrographs also present a heterogeneous pore distribution, with sizes from 14 nm to 200 nm, and a significant abundance close to 50 nm (Figure 4).

N_2_ adsorption isotherms and SEM images showed that COFs synthesized in this work have the three types of pores microporous, mesoporous, and macroporous.

#### 3.1.6. Raman Spectroscopy 

**Figure 5:**
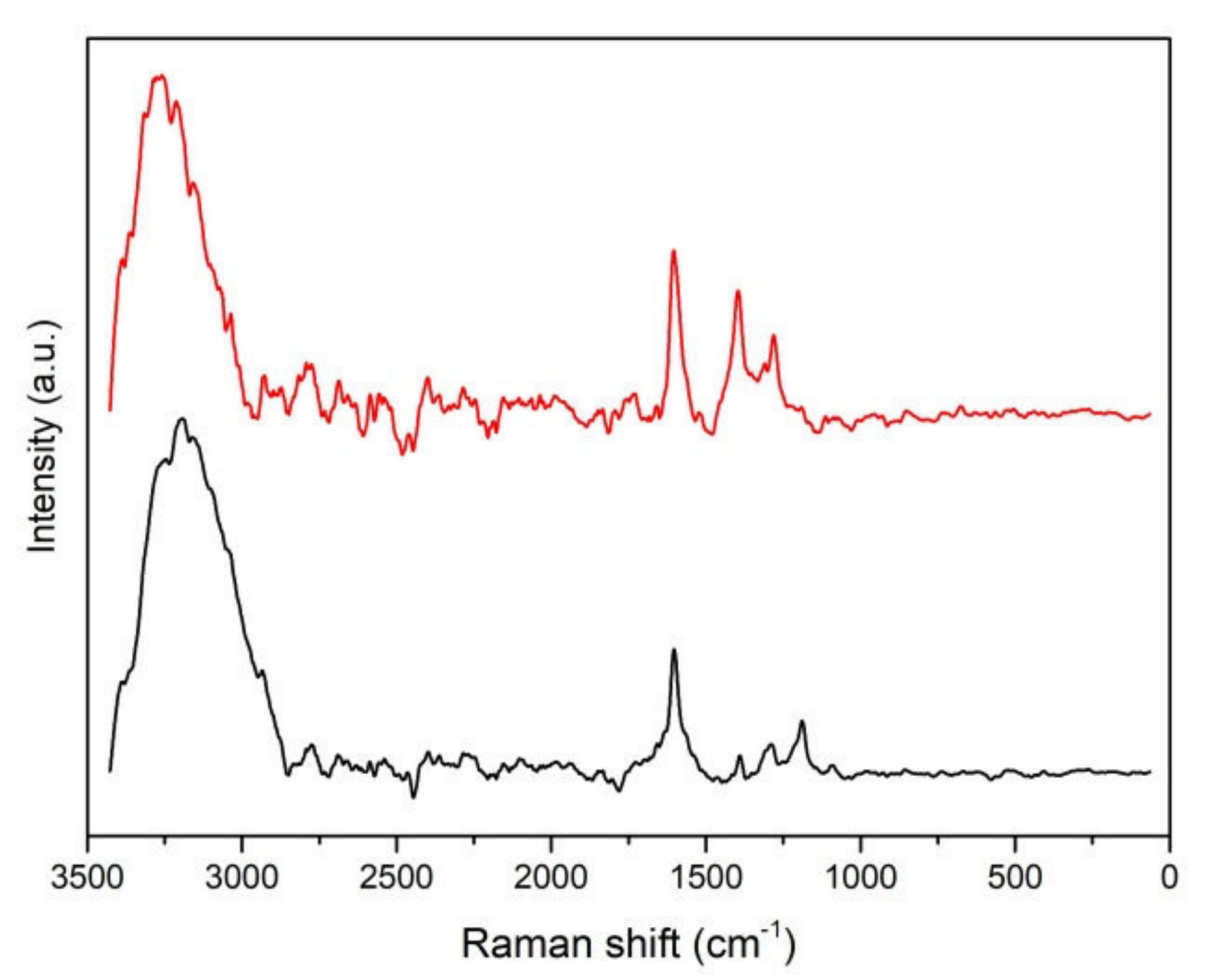
Raman spectra of TFP-BZ (black) and TFP-DMBZ (red).

These COFs presented significant interferences in the Raman spectra, so it was necessary a line base substrate and a curve smoothing. Although the final spectra obtained present no good resolved bands, nevertheless, there are two characteristics bands of these COFs. The first is the O-H stretch at 3191 cm^−1^ of TFP-BZ and 3269 cm^−1^ of TFP-DMBZ. The other signals are the C=C stretch from organic aromatic compounds at 1606 cm^−1^ of TFP-BZ and 1603 cm^−1^ of TFP-DMBZ (Figure 5). Additionally, there are two weak bands (Figure 5, red line) at 1397 cm^−1^ and 1278 cm^-1^ corresponding to the methyl rock of the 3,3-dimethylbenzidine fragment.

### 3.2. Films Characterization

**Figure 6:**
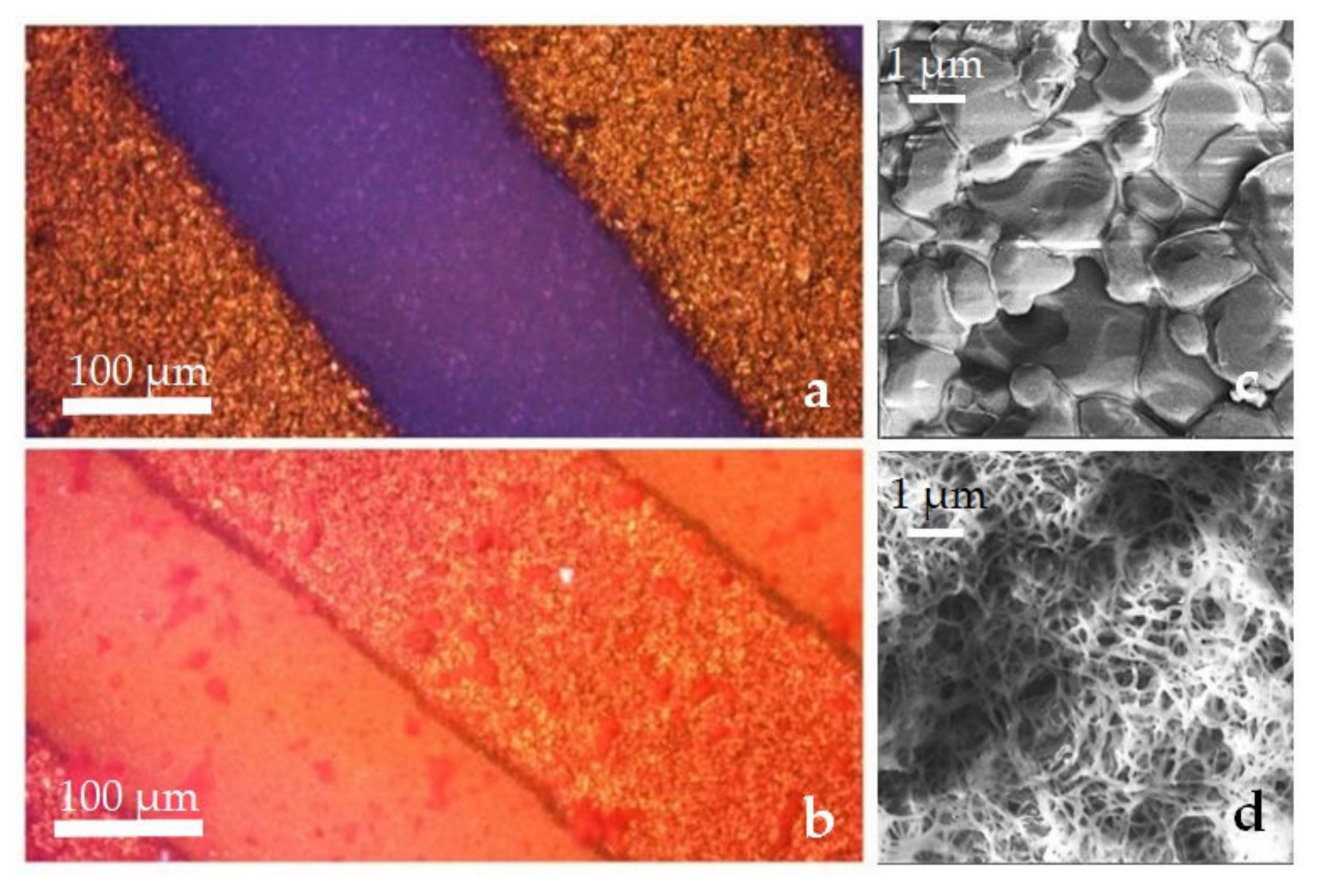
(**a**) Amplification of the pristine interdigitated region of IDE. (**b**) Amplification of TFP-BZ-IDE. (**c**) SEM image of the pristine ceramic region of IDE. (**d**) SEM image of TFP-BZ-IDE on the ceramic region of IDE.

As it was described in the synthesis section (Section 2.2), the COFs films were deposited on Au IDEs. Figure 6a is an amplification of the interdigitated region of the pristine electrode, which makes possible a visual comparison with the COF film (Figure 6b). It can be seen that the COF film covers the entire electrode surface, and there is some material accumulation as red dots. The SEM images evidence that there is a continuous COF film on the electrode, comparing the pristine ceramic region (Figure 6c) and TFP-BZ-IDE (Figure 6d). The COF film SEM image showed the presence of an intercross of wires forming a network morphology, similar to the COF powder previously characterized (Figure 4). In Figure 6, the TFP-BZ film is shown as an example. 

**Figure 7:**
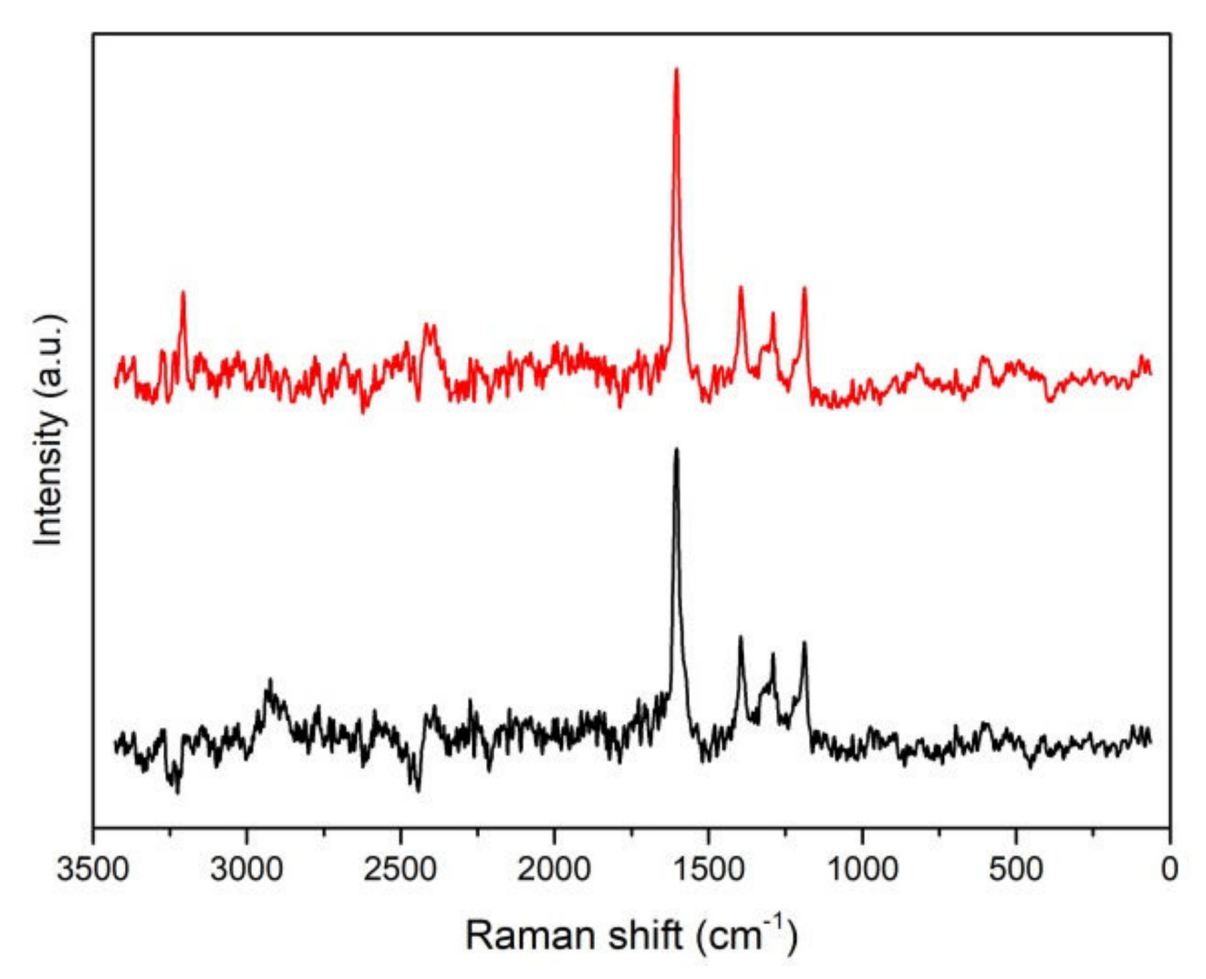
Raman spectra of TFP-BZ (black) and TFP-DMBZ (red) on Au IDEs.

The TFP-BZ-IDE and TFP-DMBZ-IDE materials were characterized by Raman spectroscopy. Again, these materials presented a signal interference making necessary baseline corrections. After that, TFP-BZ-IDE and TFP-DMBZ-IDE materials have similar bands to that of COFs powders shown previously (Figure 5). The typical C=C stretch from organic aromatic compounds was found at 1606 cm^−1^ for TFP-BZ-IDE (Figure 7, black) and 1605 cm^−1^ for TFP-DMBZ-IDE (Figure 7, red). These results confirmed the successful deposition of COFs on Au IDEs.

Unfortunately, the TFP-BZ-IDE and TFP-DMBZ-IDE did not show diffraction peaks even after employing a grazing incidence diffraction method. This is probably due to the fact that the layer of COFs deposited on the electrode has a very low thickness, or that the layer of COF has no crystalline characteristics. However, SEM images, Raman spectra, and impedance characterization (Section 3.3) showed the presence of organic material on the electrodes. This indicates that the deposition of polymers on the electrodes was successful.

### 3.3. Low Molecular Weight Amines Detection

As mentioned in Section 2.4, a potentiostat was employed for the impedance experiments. The impedance measurements showed high values around of GOhms; so, it was necessary to employ a high range of impedance potentiostat and perform the corresponding calibrations and blanks. The impedance measurements with two contact systems have been reported many times in the literature [45,46,47]. Generally, these experiments are less complex, practical, and fast to complete the electrochemical measurements. Sometimes, two contact systems in the impedance setup are a useful advantage because this makes easier the circuit model construction and its interpretation.

**Figure 8:**
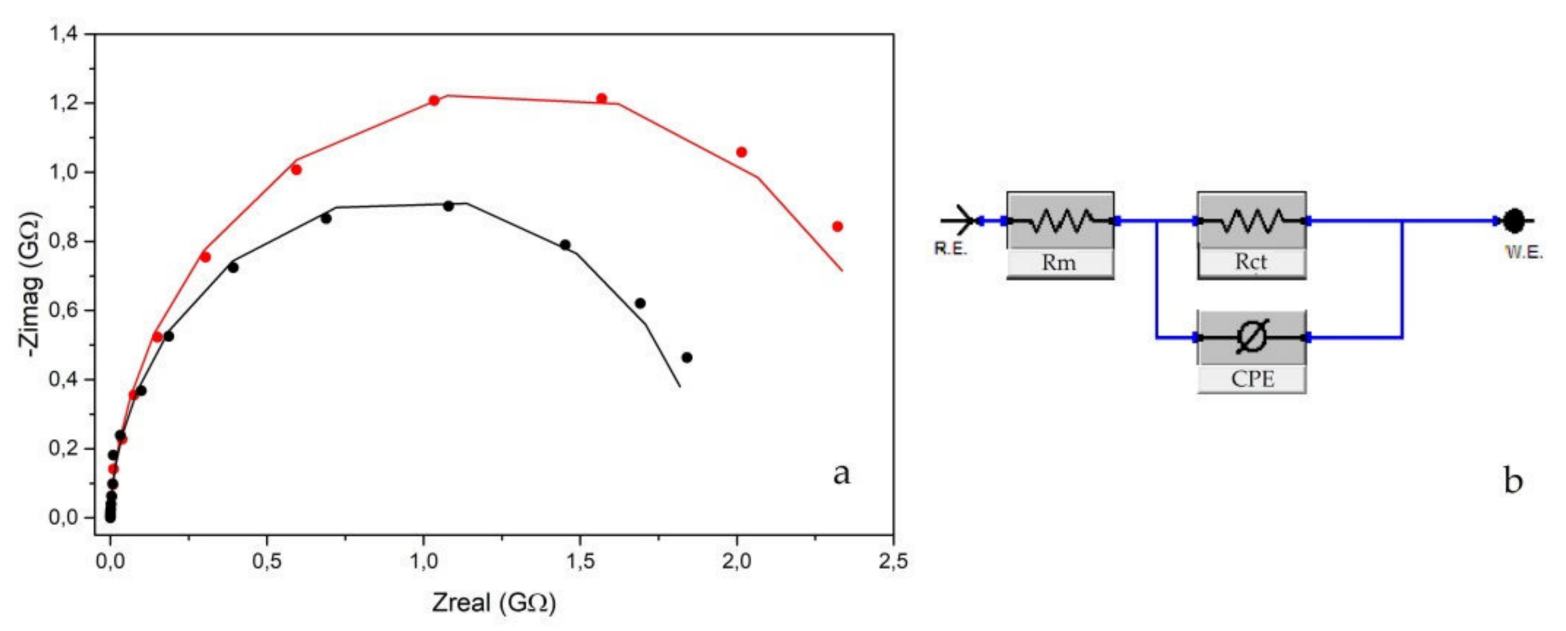
(**a**) Experimental data (dots) with circuit simulation (lines) for TFP-BZ/IDE (black) and TFP-DMBZ/IDE (red) fitting. (**b**) Electric circuit simulated.

The electrochemical characterizations of films were carried out by fitting the experimental data with circuit simulation, and it showed a good fit between experimental and simulation data (Figure 8a) for TFP-BZ-IDE (black) and TFP-DMBZ-IDE (red). The circuit used to simulate the process was a typical CPE circuit, formed by three components. Rm is the material resistance, Rct is charge transfer resistance, and CPE **constant phase element is a complex electrical double layer** (Figure 8b). 

In all experiments, the magnitude of the impedances was in GOhms (GΩ) range. The simulations showed that the most significant contribution to these impedances comes from Rct, while Rm and CPE showed less contribution to the total impedance value. These results suggested that the charge transfer between surfaces limits the electrical conduction process.

**Figure 9:**
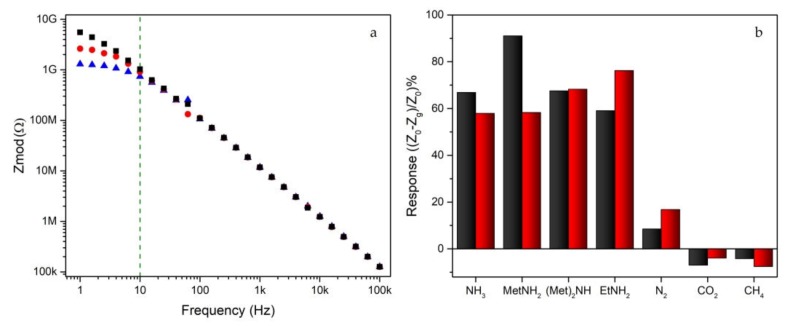
(**a**) Bode plots to TFP-BZ under 0.00 ppm (black), 1.10 ppm (red), and 2.21 ppm (blue) of ammonia. (**b**) Selectivity plot for TFP-BZ (black) and TFP-DMBZ (red).

For low molecular weight amine detection, the impedance experiments were measured under different amine concentrations and the changes in the Bode and Nyquist plots were observed. The Bode plots allow seeing the frequency region where amines generate impedance change. As an example, the Bode plots of TFP-BZ-IDE exposed to 0.00 ppm (black), 1.10 ppm (red), and 2.21 ppm (blue) of ammonia are presented in Figure 9a. Since at frequencies below 10 Hz, there were significant changes in impedance (green dashed line in Figure 9a) when changing the ammonia concentration, 1.58 Hz was chosen as the frequency for the sensing experiments with the low molecular amines.

Initially, the impedance response was studied for each amine, recording 10 points from 0.00 ppm to 44.15 ppm. For the selectivity plot (Figure 9b), 8.83 ppm amine concentration was selected to measure the impedance at 1.58 Hz. This concentration was selected because it is important to understand the behavior of the sensor at low concentrations. The four amines mentioned above were evaluated, and also, three possible interference gases (nitrogen, carbon dioxide, and methane). The TFP-BZ-IDE and TFP-DMBZ-IDE materials impedance responses were around five times greater than those found for the interference gases, showing that the TFP-BZ-IDE and TFP-DMBZ-IDE systems present significant selectivity toward the detection of amines. Although there is not a clear trend according to the chemical nature of the amine, all responses had high values of around 70%. These results showed that amine vapors affect the TFP-BZ-IDE and TFP-DMBZ-IDE impedance. No trend was seen in amine behavior, maybe because the critical factor for the COF-IDE system impedance is the charge transfer resistance (Rct), and these resistances are very similar between the COFs synthesized in this work. The impedance experiments in this work were carried out in static conditions; each measurement was made after a time for stability (20 s), and changes in the response above the same concentration were not observed. However, for employing this COF film like a sensor in future works, the measurement should be done in dynamic conditions; this way, it would be possible to evaluate the reproducibility of the sensor and how amine vapors adsorption and desorption are. 

**Figure 10:**
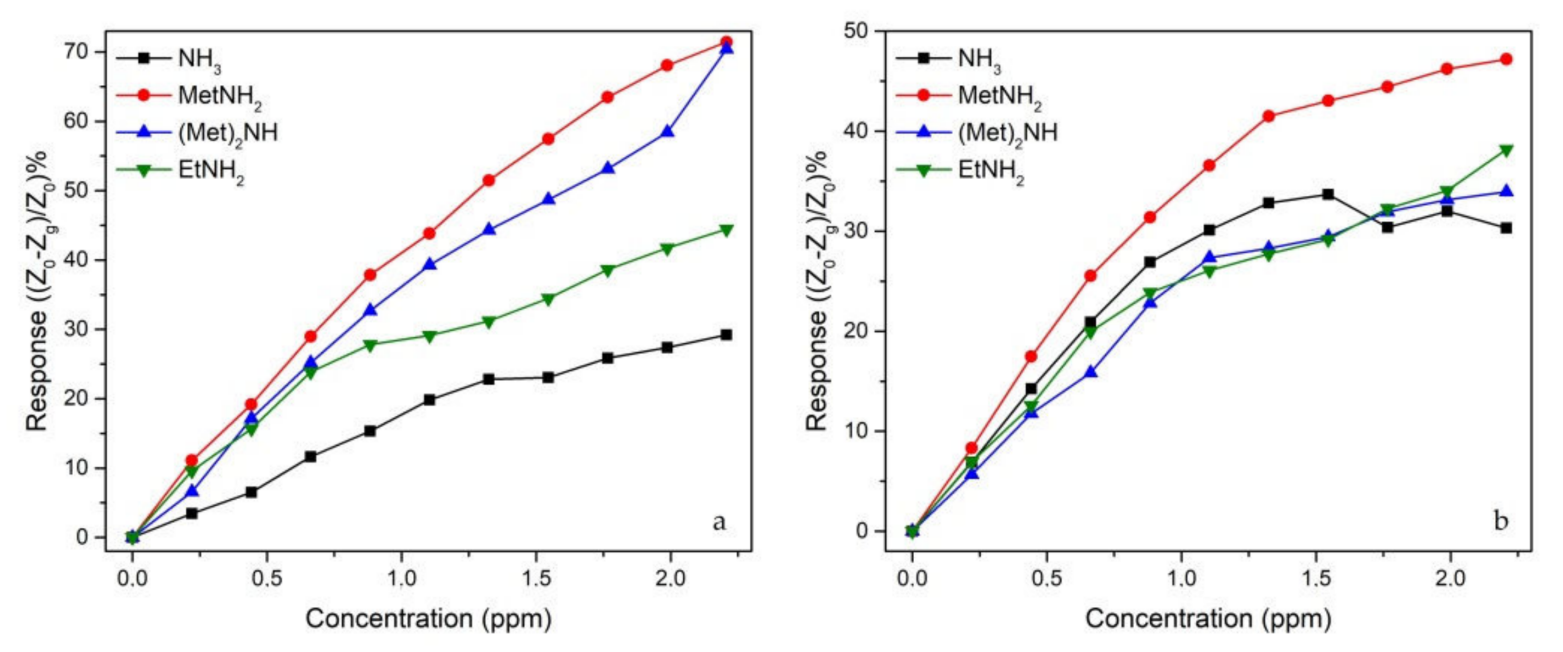
Impedance changes by different vapors amines. TFP-BZ (**a**) and TFP-DMBZ (**b**).

Additionally, the impedances changes at low amine concentrations, from 0.00 ppm to 2.21 ppm, were studied. Generally, at very low concentrations (under 0.88 ppm), the impedance responses showed a linear trend, however above 0.88, the impedance changes decreases, showing a saturated electrode behavior. The TFP-BZ-IDE (Figure 10a) showed lower impedance changes for ammonia and ethylamine, while the impedance changes by methylamine and dimethylamine are significant. On the other hand, TFP-DMBZ-IDE (Figure 10b) showed similar impedance responses for ammonia, dimethylamine, and ethylamine, whereas the methylamine change is notorious. Saturation behaviors more critical in TFP-DMBZ-IDE were observed, suggesting that this COF film has high sensitivity and could be used in concentrations approximately below 1 ppm. Nevertheless, the linear regions achieved in this work are very promising for developing a detection and quantification system for these amines.

For future work, we plan to evaluate the COFs impedance changes under a mixture of gases, between amines and interference gases. In a real application, the sensor may be under an environment with different gases at the same time. This could generate signal interference as reported in the literature [48,49].

## 4. Conclusions

The impedimetric experiments of COFs TFP-BZ-IDE and TFP-DMBZ-IDE showed that the most significant impedance contributions were from the films’ charge transfer resistances. A selectivity detection of low molecular weight amines was observed, with impedance changes around 70%. Vapor amines were detected in low concentration ranges below two ppm with a linear relationship between amine concentration and impedance responses. Therefore, these COF films are promising materials to develop a chemosensor for the detection of low molecular amines.
